# A descriptive study on diagnostic timelines, and factors influencing delayed diagnosis among adult head and neck cancer patients at Uganda cancer institute

**DOI:** 10.1186/s12957-024-03407-4

**Published:** 2024-05-16

**Authors:** Fiona Lalango, Fiona Kabagenyi, Amina Seguya, Richard Byaruhanga, Jeff Otiti

**Affiliations:** 1 Department of Ear, Nose and Throat, Moroto Regional Referral Hospital, P.O. Box 12, Moroto, Uganda; 2 Department of Ear, Nose and Throat, Makerere University College of Health Sciences, P.O. Box 7072, Kampala, Uganda; 3 Department of Ear, Nose and Throat, Mulago National Referral Hospital, P.O. Box 7051, Kampala, Uganda; 4 Department of Ear, Nose and Throat, Uganda Christian University, P.O. Box 4, Mukono, Uganda; 5 Surgical Oncology Division, Uganda Cancer Institute, P.O. Box 3935, Kampala, Uganda

**Keywords:** Time to diagnosis, Delay in diagnosis, Head and neck cancer, Uganda cancer institute

## Abstract

**Background:**

Many patients with head and neck cancer (HNC) often present with advanced disease. This may result from delay in deciding to seek care, delay in reaching the healthcare facility and or delay in accessing care in the healthcare facility. We therefore set out to determine the time to definitive diagnosis and factors associated with delayed diagnosis among patients with HNC at the Uganda Cancer Institute (UCI).

**Methods:**

A cross-sectional study was conducted at UCI, patients with HNC were recruited. An interviewer administered questionnaire was used to collect data on sociodemographic factors and clinical characteristics, including timelines in months, from symptom onset to deciding to seek care, to reaching the health care facility and to definitive diagnosis. Multivariate Poisson regression analysis was used to calculate odds ratios (ORs) for the factors of association with delayed diagnosis.

**Results:**

We recruited 160 HNC patients, and 134 patients were analyzed. The median age was 49.5 years (IQR 26.5), 70% (94 of 134) were male, 48% (69 of 134) had below secondary school education, 49% (65 of 134) had a household income < 54 USD. 56% (76 of 134) were sole bread winners, 67% (89 of 134) had good access road condition to the nearest health unit and 70% (91 of 134) presented with tumor stage 4. Median time from onset of symptoms to definitive diagnosis was 8.1 months (IQR 15.1) and 65% (87 of 134) of patients had delayed diagnosis. Good access roads (aOR: 0.26, *p* = 0.006), secondary school education (aOR: 0.17, *p* = 0.038), and household income > 136 USD (aOR: 0.27, *p* = 0.043) were associated with lower odds of delayed diagnosis. Being the sole bread winner (aOR: 2.15, *p* = 0.050) increased the odds of delayed diagnosis.

**Conclusion:**

Most of HNC patients (65%) at UCI had delayed diagnosis. A national care pathway for individuals with suspected HNC should be established and consider rotation of Ear, Nose and Throat surgeons to underserved regions, to mitigate diagnostic delay.

## Background

Head and neck cancer (HNC) refers to primary cancers of the nose, paranasal sinuses, nasopharynx, oropharynx, hypopharynx, larynx, and oral cavity. The global incidence of HNC is 8% [1] while in Uganda, an incidence of 5% is reported [2]. About 90% of HNC are squamous cell carcinoma on histopathology [3], males are more affected than females [4, 5] and the male-to-female ratio defers depending on the site involved [6].

About 51 to 70% of patients with HNC have advanced disease i.e., stage III and IV at the time of diagnosis in low-, middle and high-income countries [7–9]. The interval from the onset of symptoms to diagnosis ranges from 3 months [10] to several years [7]. Different timeframes for delayed diagnosis have been employed, such as one month [11], three months [12], and six months [13].

Various models analyze the delays in diagnosis and treatment, including the Andersen model of total patient delay [11], the three-delay model [12], and the World Health Organisation barriers to early cancer diagnosis and treatment [13]. The three-delay model developed by Thaddeus and Maine analyzes delay in three categories [12]; delay in deciding to seek care, a delay in reaching the care facility, and a delay in receiving appropriate care [8]. This model has been widely used in obstetrics and newborn care, with scanty utility in HNC [14]. This model can be adapted to and can be used to analyze time points at which delayed diagnosis occurs in HNC.

Delay in deciding to seek care may be attributed to factors such as age [5] and proximity to healthcare facilities [15]. Additionally, lack of awareness about HNC, financial limitations, and the educational level of patients and their attendants contribute to this delay [5]. Individuals with formal education were found to seek care sooner [5], and those with family support tended to seek healthcare earlier [23, 24]. Lifestyle habits, such as tobacco use, have also been associated with diagnostic delays [16].

Delays in reaching the health care facility may be influenced by the cost of transport, the condition of roads, and use of alternative medicine. Patients who live far from the health facility tend to have diagnostic delays [17]. Additionally, about 55% [18] of cancer patients in our setting use alternative medicine. Contradictory findings exist in the literature concerning the association of alternative medicine with diagnostic delays. Whereas a study done in Rwanda found an association [15], a study in Nepal reported no discernable link between alternative medicine use and delay in cancer diagnosis [15].

Delay in receiving appropriate care at the level of the healthcare system may arise from misdiagnosis among health workers in lower health centers and delayed referral [7]. Delayed completion of diagnostic investigations, such as cancer staging CT scans [10], and delays in histopathological diagnosis may result from the requirement for invasive procedures like biopsy under anesthesia (29), leading to overall diagnostic delays.

Delays in HNC diagnosis are linked to cancer stage progression [21], recurrence [22], diminished quality of life [21], and reduced survival [21]. Unpublished Uganda Cancer Institute (UCI) data reveals a more than twofold increase in HNC cases over the last 5 years, with 8 out of 10 patients diagnosed with advanced disease. The main objective of this study was to assess the time to diagnosis, with a secondary focus on identifying the factors contributing to delayed diagnosis in HNC patients at UCI.

## Methods

### Study aim

The main aim of this study was to assess the time to diagnosis, with a secondary focus on identifying the factors contributing to delayed diagnosis in HNC patients at UCI.

### Study design and period

This was an institution-based cross-sectional study conducted from October 2022 to February 2023. The study was approved by Makerere University School of Medicine Research and Ethics Committee and that of Uganda Cancer Institute (UCI).

### Study setting

Uganda has a population of 41 million, about half of the population is below 14 years and only 27% of the population are urban dwellers. Gross enrollment into secondary schools stands at only 30%, and 30% of the population live below the poverty line. Median monthly household income is low (< 60USD) [19].

This single center study took place at UCI in Kampala, central Uganda. UCI is a public, tertiary cancer training, research, and management center serving Uganda’s entire population and receiving referrals from surrounding countries such as South Sudan and the Democratic Republic of Congo. About 400 patients with a new diagnosis of HNC are seen at UCI annually.

Patients with HNC are reviewed in a formal head and neck cancer tumor board. This Tumor board consists of two head and neck surgeons, a medical oncologist, medical oncology fellow, a nurse, a radiologist, a radiation oncologist among others. Laboratory services including histopathology are free of charge. Patients however have to make out of pocket payments for imaging services such as computerized tomography scans which many of them cannot afford.

### Study population

Patients ≥ 18 years with a histopathological diagnosis of HNC at UCI between October 2022 and February 2023 were recruited.

### Eligibility criteria

All patients aged ≥ 18 years with a histopathological diagnosis of HNC, regardless of whether they were newly diagnosed, on treatment, or in follow-up, were included. The included sites were; the nose, paranasal sinuses, nasopharynx, oral cavity, oropharynx, larynx, and hypopharynx. Exclusions comprised patients too weak to participate or with whom effective communication was not possible.

### Sampling size techniques and sampling procedure

The sample size for the primary aim (*n* = 104) was determined using Andrew Fisher’s (1935) formula, based on a study by Kassirian et al. This was a single institution cross-sectional study in which 102 HNC patients participated. The mean (SD) from onset of symptoms to review in tumour board was 15.07 months (± 31.54) [23].

For the primary aim, we used the sample size estimation for a single mean in one group

$$\varvec{N}=\left(\frac{\varvec{Z}(1-\frac{\propto }{2})\varvec{*}\varvec{S}}{\varvec{d}}\right)\bigwedge 2$$

Where;

- N was the sample size estimated.
- Z 1-α/2 was the standard normal value corresponding to the level of significance = 1.96.
- S was the standard deviation.
- d was the precision of the mean which will be determined as 3% of the mean.

$$N=\left(\frac{1.96X31.54}{0.03X15.07}\right)\bigwedge 2$$

The derived sample size was 18,697.

This was a very large number and could not be obtained. Using the raw data at UCI where approximately 40 new and 20 follow-up head and neck cancer patients are seen monthly, a total of 180 in three months was got. This duration of three months was the initial study duration that was considered to down size the sample size.

$$\varvec{S}=\frac{\varvec{N}}{1+\raisebox{1ex}{$\varvec{N}$}\!\left/ \!\raisebox{-1ex}{$\varvec{p}\varvec{o}\varvec{p}\varvec{u}\varvec{l}\varvec{a}\varvec{t}\varvec{i}\varvec{o}\varvec{n} \varvec{s}\varvec{i}\varvec{z}\varvec{e}$}\right.}$$

Where;

- N was the non-adjusted sample size.
- S was the adjusted sample size.
- The population was the expected number of subjects within the time frame.

Applying the above formula

$$\varvec{S}=\frac{18,697}{1+\raisebox{1ex}{$18,697$}\!\left/ \!\raisebox{-1ex}{$180$}\right.}$$

S = 104 participants.

For the secondary aim; factors associated with delayed diagnosis

$$\begin{array}{l}N=\\\frac{{\left({Z}_{\propto }\surd P\left(1-P\right)\left(\raisebox{1ex}{$1$}\!\left/ \!\raisebox{-1ex}{${q}_{1}$}\right.+\raisebox{1ex}{$1$}\!\left/ \!\raisebox{-1ex}{${q}_{2}$}\right.\right)+{Z}_{\beta }\surd {P}_{1}\left(1-{P}_{1}\right)\left(\raisebox{1ex}{$1$}\!\left/ \!\raisebox{-1ex}{${q}_{1}$}\right.\right)+{P}_{2}\left(1-{P}_{2}\right)\left(\raisebox{1ex}{$1$}\!\left/ \!\raisebox{-1ex}{${q}_{2}$}\right.\right)\right)}^{2}}{{\left({P}_{1}-{P}_{2}\right)}^{2}}\end{array}$$

Where;

- Zα/2 was the standard normal value corresponding to the level of significance (e.g., for a confidence level of 95%, α is 0.05 and the critical value is 1.96),

Zβ was the standard normal value corresponding to the power of the study (e.g., for a power of 80%, β was 0.2 and the critical value was 0.84), Considering knowledge of head and neck cancer as the factor of interest from a study done by Tromp et al. in Netherlands [20], 

Proportion of individuals in group 1 with the outcome, p1 = 0.326,

- Proportion of individuals in group 2 with the outcome (p2) = 0.559.
- Proportion of participants in group 1 (q1) = 0.331 and.
- proportion of participants in group 2 (q2) = 0.669.

P = p1q1 + p2q2

Based on the above formula, the calculated sample size was 159 participants.

Eventually, the sample size for objective 2 = 159 was taken as the minimum sample size because it was bigger than 104.

### Study variables

#### Dependent variables

The primary outcome was the time to definitive diagnosis of HNC patients. This was calculated from the time of onset of signs and symptoms to the time the patients obtained the definitive histopathological diagnosis, measured in months. The cut-off for delay was greater than 6 months, cross-verified with patients’ files. In cases of disparity, interview-provided timing was utilized. We chose 6 months to be accommodative because Ear Nose and Throat Specialists are not easily accessible to patients in our setting. Two studies in in similar settings used 6 months. One was by Pace et al. who found a cut off of 6 months to give meaningful results and the other was by Adeyi et al.

#### Other time intervals measured included

The time from onset of symptoms to deciding to seek care, the time from deciding to seek care to the first medical visit and the time from the first medical visit to the definitive diagnosis. The cut-off for each interval was more than two months. In cases where the patient could not recall the precise date, the 1st, 15th, or 30th of the month was assigned based on whether they reported symptoms at the beginning, middle, or end of the month, respectively or the date of the next week day if any of the dates fell on a weekend.

#### Independent variables

Socio-demographics and patient related factors included age, sex, marital status, level of education, household income, condition of access roads and tumour stage (using American Joint Committee on Cancer AJCC 8th edition). Healthcare system factors included use of herbal treatment, cadre of healthcare worker first visited, referral information, affordability of laboratory investigations, affordability of histopathological investigations, affordability of computerized tomography scan and histopathological diagnosis before UCI.

### Study procedure and tool

Patients with a HNC diagnosis were identified from the Head and Neck Tumour Board (HNTB) at UCI. Consecutive sampling was used. Lists of eligible patients were generated by the three nurses attached to the HNTB and review clinics; they doubled as our research assistants. The lists generated were for patients who had come to either attend the HNTB or review clinics. Written informed consent was obtained and interviewer administered questionnaire developed by the researchers and the patient’s medical records were used to collect data. The research questions were informed by findings from published similar studies observations made from the day-to-day running of the tumor board. They were pretested and fine-tuned prior to use.

### Data quality control

The study questionnaire underwent a pilot to enhance its utility before initiation. Research assistants were thoroughly trained before data collection. Their responsibilities included identifying eligible patients, generating lists, and obtaining medical files. The Principal Investigator (PI) verified patients from the list, enrolled them in the study, and administered the questionnaire to prevent data duplication.

### Statistical analysis

Statistical analysis was done using program R version 4.3.0. Continuous variables were analyzed using mean, median, and range as appropriate and these are presented in tables and figures. Delayed diagnosis was considered as a period of more than 6 months from symptom onset to definitive diagnosis and is presented in months. Multivariate Poisson regression was used to calculate crude and adjusted odds ratios. Factors were considered to be statistically significantly associated with delayed diagnosis if *p* > 0.05.

## Results

### Socio-demographic characteristics and other patient related factors

Out of the 178 patients screened for this study, 18 were excluded. Ultimately, 160 patients were included in the study. Among the recruited patients, those with salivary gland [14], thyroid [6] and others [6] were excluded from the analysis due to differences in tumor biology. Hence, the results provided pertain to 134 patients, with 70% (94 or 134) being male (Tabe 1). The median age was 49.5 IQR 26.5 years. Approximately 51% (69/134) of the patients had attained at least a secondary school education, 49% (65/134) had a monthly household income of < 54 USD (United States Dollars) and about 70% (91/134) of the patients had stage IV cancer at diagnosis (See Table 1).

**Table 1:** Socio-demographic characteristics and other patient factors

Variable		Frequency (*n* = 134)	Percentage (%)
Sex	Female	49	30
	Male	94	70
Age	18–50	69	51.5
	51–64	40	29.9
	≥ 65	25	18.6
Marital status	Married	93	69.4
	Unmarried	41	30.6
Level of education	None	13	9.7
	Primary	52	38.9
	Secondary	32	23.8
	Tertiary	37	27.6
Household income (USD)	< 54	65	48.5
	54–136	37	27.6
	> 136	32	23.9
Paid time off work	No	104	78.2
	Yes	30	21.8
Sole bread winner	No	58	43.3
	Yes	76	56.7
Affordability of	No	31	23.2
transport	Yes	103	76.8
Access road	Bad	45	33.5
condition	Good	89	66.5
Smoking status	Current	0	0
	Former	38	28.4
	Never	96	71.6
Signs and symptoms due to cancer*	No	124	92.5
	Yes	10	7.5
Tumor stage	1	6	4.6
	2	12	9.1
	3	22	16.8
	4	91	69.5
Tumor site	Sinonasal	20	14.9
	Nasopharynx	40	29.9
	Oropharynx	10	7.5
	Hypopharynx	5	3.7
	Larynx	27	20.1
	Oral cavity	32	23.9

(*This statement was in respect to whether the patients were able to associate their signs and symptoms with cancer)

### Healthcare system related factors

Herbal medicine was not utilized by 59% of the patients, and 71% faced challenges affording a CT scan (See Table 2).

**Table 2:** Healthcare system related factors

Variable		Frequency (*n* = 134)	Percentage (%)
Use of herbal treatment	No	79	59
	Yes	55	41
Patients referred	No	42	33.3
	Yes	81	66.7
Previous	No	12	9
histopathology	Yes	122	91
Anesthesia prior to	Yes	42	31.5
biopsy	No	92	68.5
Laboratory affordability	No	5	3.8
	Yes	129	96.2
Histopathology	No	55	41
affordability	Yes	79	59
Ultra sound scan	No	26	19.4
affordability	Yes	108	71.6
Affordability of CT	No	95	70.9
	Yes	39	21.1

**Fig. 1:**
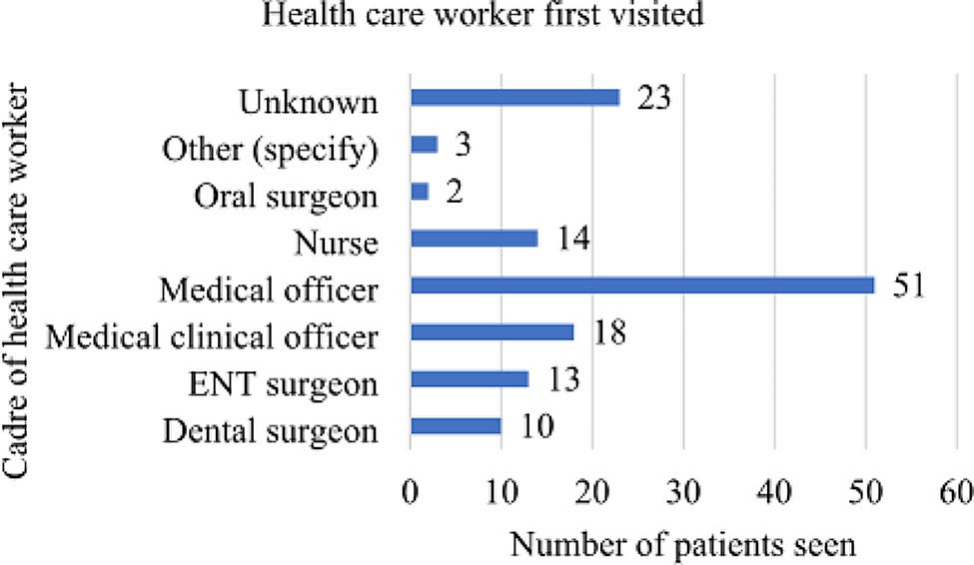
A bar graph showing the cadre of healthcare worker first visited

**Fig. 2:**
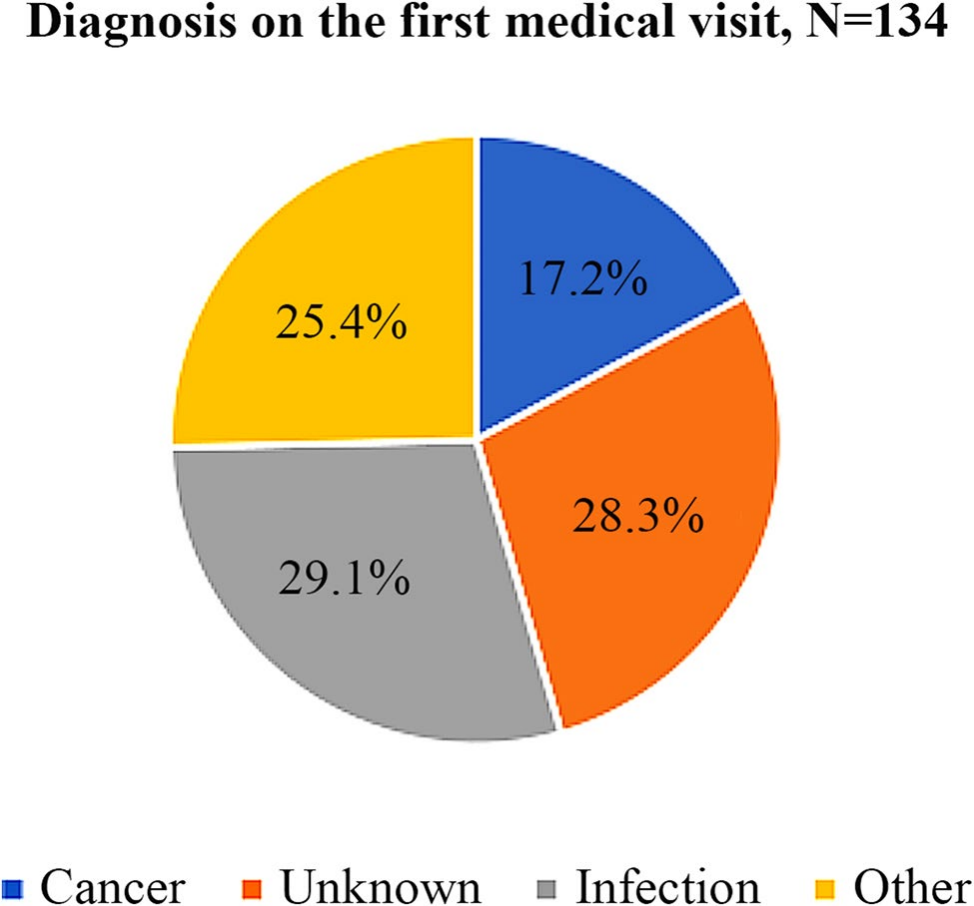
A pie chart showing the diagnosis on the first medical visit

The predominant cadre of healthcare workers visited first was medical officers, accounting for 38% (51/134) of the cases. Only 9.7% (13/134) of the patients had their first medical visit with an Ear, Nose, and Throat surgeon (See Fig. 1). On their initial medical visit, only 17% of the 134 patients were suspected to have cancer (see Fig. 2).

### Time to diagnosis

The median time to definitive diagnosis was 8.1 months. Of the other time intervals measured, within facility delay was the longest with a median of 5.2 month. There were variations by tumor site with the larynx having the longest median of 11.6 months and the hypopharynx and the shortest median of 6.2 months (See Table 3).

65% of the patients (87/134) had delayed diagnosis.

**Table 3:** Timelines in months

Timelines (in months)	Median (IQR)	Range
The onset of symptoms to the decision to seek care (Delay 1)	2(0.5-7)	0–50
The decision to seek care to first medical visit (Delay 2)	0.03(0-0.1)	0-31.5
First medical visit to the definitive diagnosis (Delay 3)	5.2(3.2–12)	0-102
*Total time interval* The onset of symptoms to the definitive diagnosis	8.1 (5.3–23.2)	1.1-263.5
Time by tumour site* Sinonasal	8.4	1.1–45
Nasopharynx	9	1.6–64.6
Oropharynx	10.7	2.1–64.6
Hypopharynx	6.2	4–27
Larynx	11.6	4–62
Oral cavity	7.5	1.4–119

IQR is interquartile range

(*This was in respect to total delay)

**Fig. 3:**
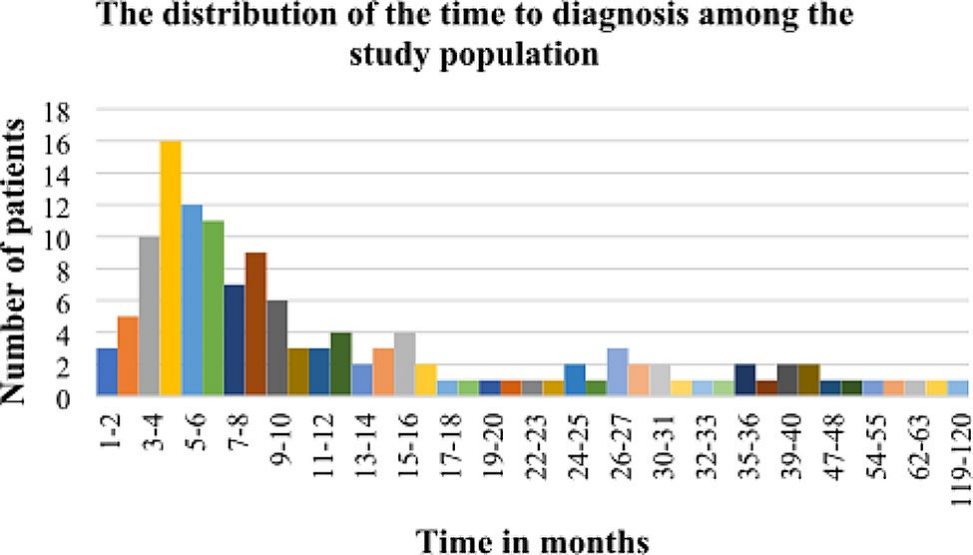
Histogram showing the distribution of the time to diagnosis among the study population

The proportion of patients who had the definitive diagnosis ≤ 3 months following the onset of symptoms was 5.9%, > 3 to ≤ 6 months 25.1%, and > 6 months 64.9%. The overall distribution of the time to diagnosis among the study population is shown in Fig. 3.

### Factors associated with delayed diagnosis among HNC patients

At bivariate analysis, the level of education, being the sole bread winner, household income, the condition of access roads and affordability of histopathology had a *p*-value < 0.2. These variables were analyzed using poison multivariate regression analysis.

Statistically significant factors included the level of education, being the sole breadwinner, household income, and the condition of access roads.

The odds of delayed diagnosis among those who had a secondary school education were 0.17 (_a_OR=0.17, 95% CI 0.03–0.91). Patients who were the sole bread winners had higher odds of delayed diagnosis 2.15 (_a_OR = 2.15, 95% CI 1.00-4.39). Patients with household income > 136 USD had lower odds of delayed diagnosis 0.27 (_a_OR = 0.27, CI 0.08–0.96). The odds of delayed diagnosis were lower in patients who had good access roads 0.26 (_a_OR=0.26, CI 0.01–0.68) (See Table 4 below).

**Table 4:** Factors associated with delayed diagnosis

Variable		No delay (*n* = 47)	Delay (*n* = 87)	_c_OR (95% CI)	_a_OR (95%CI)	*P*-value
Level of	None	3(6.3)	10(11.5)	1		
education	Primary	13(27.7)	39(44.8)	0.90(0.21–3.78)	0.50(0.11–2.34)	0.375
	Secondary	17(36.2)	15(17.2)	0.26(0.06–1.15)	0.17(0.03–0.91)	0.038*
	Tertiary	14(29.8)	23(26.4)	0.49(0.12–2.10)	0.44(0.07–2.83)	0.39
Sole bread winner	No	26(55.3)	32(37.2)	1		
	Yes	21(44.7)	54(62.8)	2.03(0.99–4.14)	2.15(1.00-4.93)	0.05*
household	54–136	9(19.2)	28(32.2)	1		
Income (USD)	< 54	20(42.6)	45(51.7)	0.72(0.29–1.81)	0.44(0.15–1.27)	0.13
	> 136	18(38.2)	14(16.1)	0.25(0.09–0.70)	0.27(0.08–0.96)	0.043*
Did you think	No	45(95.7)	79(90.8)	1		
You had cancer	Yes	2(4.3)	8(9.2)	2.28(0.46–11.20)		
Herbal	No	29(61.7)	50(57.5)	1		
treatment	Yes	18(38.3)	37(42.5)	1.19(0.58–2.46)		
Smoking	Former	12(25.5)	28(23.7)	1		
	Never	35(74.5)	90(76.3)	0.80(0.36–1.79)		
Affordability	No	10(21.3)	22(25.3)	1		
Of transport	Yes	37(78.7)	65(74.7)	0.85(0.36-2.00)		
Access road	Bad	8(17.0)	37(42.5)	1	1	
Condition	Good	39(83.0)	50(57.5)	2.28(0.12–0.66)	0.26(0.01–0.68)	0.006*
Affordability of histopathology	No	15(31.9)	40(46)	1		
	Yes	32(68.1)	47(54)	0.55(0.26–1.16)		

Where _c_OR is the crude odds ratio and aOR is adjusted odds ratio

## Discussion

Conducted over five months at UCI, our study had dual objectives: firstly, to ascertain the duration from symptom onset to definitive diagnosis among HNC patients, and secondly, to identify factors linked to their delayed diagnosis. Our study revealed a median time to definitive diagnosis of 8.1 months (IQR 15.1), with 65% (87/134) experiencing delayed definitive diagnosis. Within facility delay was the longest with a median of 5.2 months (IQR 8.8). Increased odds of delay were associated with being the sole breadwinner, while reduced odds were observed with secondary education, a higher household income and good access road conditions to the nearest health unit.

Compared to a UK study where the time from symptom onset to definitive diagnosis was 3 months, largely due to patient-related delays [21], our study observed a longer duration (> 8 months). The healthcare system likely contributed significantly to this delay with a median of 5.2 months (IQR 8.8). Misdiagnosis of patients by initial healthcare providers was high at 82%. In the study by Onyango et al., 77% of the patients were managed with unspecified medication and only 16% had a biopsy done [7]. This is comparable to our study where 82% of the patients were misdiagnosed and about 17% suspected to have cancer by the first healthcare workers they visited. Issues of mismanagement were also reported by Alho et al. [22], suggesting inadequate patient management and delayed referrals by non-specialist healthcare providers.

Variations in median delay to definitive diagnosis were noted by subsite, with laryngeal cancers having the longest median delay of 11.6 months and hypopharyngeal cancers having the shortest median of 6.2 months. This could be because of the difference in signs and symptoms based on the sites which would influence the decision to seek healthcare. Patients delay to seek healthcare when they do not perceive their signs and symptoms to be serious [23]. A study by Jensen et al. found a reduced likelihood of cancer patients being referred if the primary care physician did not consider the signs and symptoms concerning [24]. Hypopharyngeal cancers which often present with swallowing problems may prompt earlier referral by healthcare workers visited than laryngeal cancer which often present with hoarseness of voice which may be considered just discomforting.

Our research revealed a 65% rate of delayed diagnosis among patients, differing from the 79% reported by Adeyi et al. in Nigeria [25], despite similar herbal medicine usage. This variance may stem from changes in health-seeking behavior over the past decade. While we did not establish a correlation between herbal medicine usage and delayed diagnosis, Pace et al. identified diagnostic delays in breast cancer patients visiting traditional healers [15]. This disparity in findings may arise from variations in assessment methodologies. Herbal treatment, a component of traditional and complementary medicine, is accessible to patients regardless of their engagement with traditional healers [26].

Our findings revealed that being the sole breadwinner was linked to increased odds of delay, with an odds ratio of 2.15 and a significance level of 0.05. This can be explained by the fact that 78% of the patients enrolled in this study were unable to take paid time off work to seek healthcare. Data from the Uganda Demographic Health Survey of 2019/2020 showed that 52% of workers had inadequate earnings and difficult work conditions that undermined their fundamental rights. Median monthly household incomes were low (< 60 USD) with 11% of households spending more than 40% of their earnings on healthcare [19]. Sole breadwinners had to prioritize providing for their families as a matter of survival. Forbes et al., found being too busy to visit the doctor was associated with delayed presentation of cancer patients, with an odds ratio of 2.3 [25]. Although linkage between this being busy work conditions and household income was not made.

Our study observed that the level of education and household income were statistically significant. Patients with secondary education had lower odds of delay with an odds ratio of 0.15 and a significance level of 0.038. This is in keeping with findings by Baishya et al. who found education to be associated with a shorter median delay [26]. Education may affect the way patients perceive their symptoms prompting them to seek healthcare earlier. Also, patients with a higher level of education are more likely have better employment conditions that enable them to have easier access to healthcare [27]. Patients with a monthly household income exceeding 136 USD exhibited reduced odds of delayed diagnosis, with an odds ratio of 0.27 and a *p*-value of 0.043. This corresponds to the results reported by Agarwal et al. [5]. A higher income allows patients to cover out-of-pocket expenses for investigations, thereby facilitating earlier diagnosis.

Notably, favorable access road conditions to the nearest health facility were linked to reduced odds of delayed diagnosis, with an odds ratio of 0.26 and a *p*-value of 0.006. Luna et al.‘s research revealed that access to well-maintained roads enhances the accuracy of diagnosis for various illnesses, particularly those affecting children and women [17]. Improved roads may attract more qualified healthcare professionals, streamline access to care, and enhance transportation of medical supplies. However, there is limited literature examining the connection between delayed diagnosis and road conditions.

Our study found no correlation between delayed diagnosis and age, sex, or marital status, consistent with the findings of Kassirian et al. [28]. This consistence of findings could be because both were single centre studies which used consecutive sampling of patients. Brouha et al. found an association between tobacco smoking and delayed HNC diagnosis [16]. The differences could be attributed to differences in study populations and design. Brouha studied patients with oral cavity, laryngeal and pharyngeal cancer. All these cancers have tobacco smoking as a known risk factor unlike our study that also included sinonasal cancer patients. Unlike Brouha, we did not quantify the amount of tobacco smoked by the patients.

No healthcare factor, such as the need for examination under general anesthesia before biopsy or the costs of histopathology, laboratory investigations, ultrasound scans, or CT scans, was associated with late delayed diagnosis at the multivariate analysis stage in this study. The lack of association at the multivariate level may be due to the fact that the specific diagnostic timeline influenced by these factors was not assessed for association in this study.

### Limitations

There was selection bias introduced when the very sick were excluded and executing the study at a single facility. Only patients who could access the facility were enrolled. Recall bias was introduced when some patients were not able to remember the necessary dates. However, medical records were used to mitigate this. Dates that could not be obtained from medical records were estimated in a standardized way.

## Conclusions

In UCI, there exists a significant burden of delayed diagnosis among HNC patients, with 65% experiencing delays. The median total delay was 8.1 months (IQR 17.9) with delay within the healthcare system contributing the most median 5.2 months (IQR 8.8). The condition of access roads emerges as the primary contributing factor, alongside household income, level of education, and sole breadwinner status, all demonstrating significance. To mitigate these, urgent measures should be taken to improve the condition of access roads leading to healthcare facilities. Continuous medical education of medical officers, dental surgeons and other cadres of healthcare workers who are first contacted by the patients to enable earlier identification. A national care pathway for patients with signs and symptoms of head and neck cancer to reduce within facility delay. Rotation of Ear, Nose and Throat surgeons to underserved regions to enable easier access by patients with signs and symptoms of HNC. Financial assistance programs targeted at patients from low-income households should be implemented to alleviate the financial barriers associated with seeking medical care.

## Data Availability

All data sets used and/or analyzed during this current study are available from the corresponding author on reasonable request.

## Abbreviations

AJCC: American Joint Committee on Cancer
HNC: Head and neck cancer
HNTB: Head and neck tumor board
PI: Principal investigator
TNM: Tumor, Lymph nodes, Metastasis
UCI: Uganda Cancer Institute
